# Cognitive modeling, ecological psychology, and musical improvisation

**DOI:** 10.3389/frobt.2023.1126033

**Published:** 2023-08-17

**Authors:** Kevin J. Ryan

**Affiliations:** Department of Philosophy, University of Tennessee, Knoxville, TN, United States

**Keywords:** improvisation, cognitive model, ecological psychology, learning intelligent decision agent (LIDA), affordances, music

## Abstract

Understanding novelty and improvisation in music requires gathering insight from a variety of disciplines. One fruitful path for synthesizing these insights is via modeling. As such, my aim in this paper is to start building a bridge between traditional cognitive models and contemporary embodied and ecological approaches to cognitive science. To achieve this task, I offer a perspective on a model that would combine elements of ecological psychology (especially affordances) and the Learning Intelligent Decision Agent (LIDA) cognitive architecture. Jeff Pressing’s cognitive model of musical improvisation will also be a central link between these elements. While some overlap between these three areas already exists, there are several points of tension between them, notably concerning the nature of perception and the function of artificial general intelligence modeling. I thus aim to alleviate the most worrisome concerns here, introduce several future research questions, and conclude with several points on how my account is part of a general theory, rather than merely a redescription of existent work.

## 1 Introduction

To understand novelty in cognition, we must account for improvisation; as philosopher Gilbert Ryle noted about improvising, “It is part of intelligence to seize new opportunities and to face new hazards; to be, in short, ‘not a tram, but a bus’” [(Ryle, 1976), 69]. Other philosophers have explored improvisation in detail, including, *inter alia*, its connection to composition and repetition, possible moral dimensions, and its relation to creativity and novelty (Brown, 1996; Gould and Keaton, 2000; Alperson, 2010; Carvalho, 2010; Hagberg, 2016; Brown, Goldblatt and Gracyk, 2018; Lewis, 2019). My focus will hereafter be on musical improvisation.

The scientific study of improvisation in music has seen major development in the past several decades. Landmark work by musician and psychologist Pressing. (1988), Pressing (1998) has left a lasting impact on cognitive accounts of musical improvisation. Another important computational account was developed by psychologist and philosopher Johnson-Laird (2002). Similarly, work in artificial intelligence has been steadily advancing. Examples of notable AI programs here include Voyager (Lewis, 2000), Magenta, and BachBot (Novelli and Proksch, 2022).

Since the early 2000s, neuroscientists have discovered important roles for different neural areas in improvisation, including (but not limited to) the left inferior frontal gyrus (IFG) and the dorsolateral prefrontal cortex (DLPFC) (For a review, see Beaty, 2015; for a conceptual model involving neural correlates, see Faber and McIntosh, 2019). Evidence has emerged for differences during solo vs. group improvisation, most notably in activations of the DLPFC. Interpretations for why these differences occur include unique requirements for creativity and monitoring in individual or collective contexts; there may also be methodological differences in participant selection and task requirements across studies (Beaty, 2015).

Another discipline to consider is ecological psychology, wherein the environment is taken to be both an inextricable part of psychological explanation and a constitutive part of cognition. A pioneer of this approach was psychologist Gibson 1966, Gibson 1979. It has further developed to include accounts of auditory cognition (Gaver, 1993a; Gaver, 1993b), music perception (Clarke, 2005), music in everyday life (DeNora, 2000, esp. Chap. 4), musical affordances (Reybrouck, 2012; Windsor and de Balzac, 2012; Krueger, 2014), and the structure of performance spaces (Burland and Windsor, 2014), among other topics. Cognitive scientist and philosopher Ashley Walton and her colleagues (Walton et al., 2015; Walton et al., 2018) have also provided similar work on dynamics and interaction in performance.

In what follows, I propose an important way to further our understanding of musical improvisation by bringing together ecological psychology, the Learning Intelligent Decision Agent (LIDA) cognitive architecture, and Pressing’s model of musical improvisation. I first introduce Pressing’s model. Second, I introduce core aspects of LIDA. Third, I present an ecological description of Pressing’s model from music theorist and lawyer Love. (2017) and, finally, I discuss several main issues about how Pressing, Love, LIDA can be connected. While there are insights for many different readers in what follows, I hope that it speaks clearly to those who are interested with an account of improvisation in cognition and action, including the similarities and differences between domain general and domain specific aspects of improvised activity.

## 2 Pressing’s model of musical improvisation

There is an expansive and growing literature for models, including theories and interdisciplinary work, of musical performance (e.g., see MacDonald and Wilson, 2020, Chap. 2, esp. 30–43, for a theoretical overview). There are likewise notable differences among traditions of cognitive modeling. For instance, classical approaches focus on the development of music starting within an agent, post-human approaches place emphasis on the flow of information from the environment into the agent, computational accounts take most, or all, of human cognition as akin to functions on computers, and embodied approaches center cognition as a dynamic dance between brain, body, and world.

Pressing had a classical approach to modeling musical improvisation^1^. The heart of his model is the referent and the knowledge base of an improviser. A referent is “a set of cognitive, perceptual, or emotional structures (constraints) that guide and aid in the production of musical materials” (Pressing, 1998, 52). Paradigmatic examples include a jazz standard or a 12-bar blues progression. Some referents are stored in external formats, utilizing musical notation and sheet music, while others are internalized. The purpose and existence of a referent will vary depending on the type of improvisational activity and other relevant considerations.

The knowledge base covers a range of information stored primarily in long-term memory. Specific elements include “musical materials and excerpts, repertoire, subskills, perceptual strategies, problem-solving routines, hierarchical memory structures and schemas, generalized motor programs, and more” (Pressing, 1998, 53). The base can further be connected with three mental representations: objects, features, and processes. Objects are specific “cognitive units,” such as a chord or gesture; features are “common parameters of multiple objects; ” processes are “changes in objects or features over time; ” and, finally, it is essential to highlight that all of these aspects interact with each other in complex ways (Dean and Bailes, 2014, 41–42).

According to Pressing, improvisors execute plans to either continue current musical events via association or break via interruption. The general forms of these choices are consistent–association maintains most/all of the aspects (e.g., movement, musical, acoustic, or other features) from previous events, while interruption breaks from them to some significant degree–but their exact functioning in performance will vary based on multiple factors. For example, a bassist may decide to play a pedal point across several measures. A continuation of the tone would be an association between the starting event (E_i_) and E_i+1_, E_i+2_, … E_i+n_, while abruptly stopping it would be an interruption between these events. Reasons for choosing continuity or interruption include acoustic, social, musical, and movement-based considerations, among others. These processes are also facilitated by an interruption tester monitoring E_i_ and a movement trigger between events (For a visual overview of Pressings model, see Fig. 7.4 in Pressing, 1988, 160).

There are two further things to highlight about Pressing’s model. First, it operates under an assumption that improvisation develops along a discrete repetition of input-process-output cycles. These cycles are musical events and event clusters. For instance, a given solo for a jazz bebop performance would be an event cluster constituted by multiple interlocking musical events (e.g., specific notes and changing chords). Second, his model is developed at a rather general level. In Pressing’s words, for instance, the model “does not spell out exactly how real-time constraints of memory and attention are to be accommodated” (1998, 56). Connecting this model to LIDA will help to address some of these downsides and further strengthen the upsides.

## 3 Learning intelligent decision agent (LIDA) cognitive architecture

**FIGURE 1:**
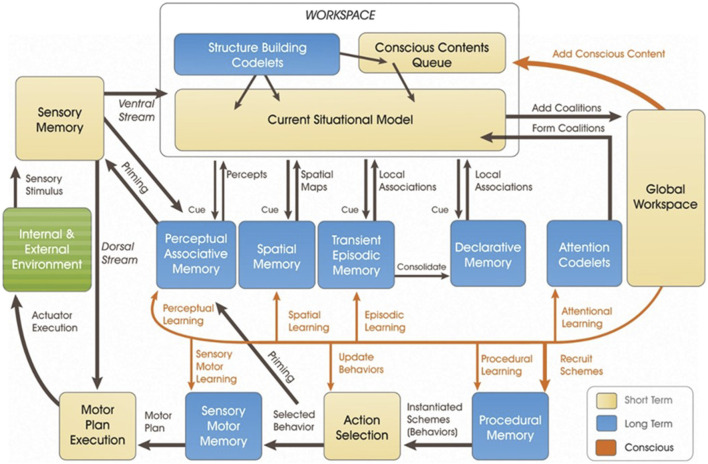
The LIDA Model Overview.

Learning Intelligent Decision Agent (LIDA) is a conceptual and partially computational systems-level cognitive model that aims to model mind (see Figure 1). Insofar as it operates at the systems-level, LIDA attempts to account for the entire span of low-level to high-level cognition. According to Stan Franklin, computer scientist and the main architect behind LIDA, a mind is a control structure of an autonomous agent (AA). AA has a technical definition of “a system situated in and part of an environment, which senses that environment and acts on it over time in accordance with its own agenda, so as it may affect what it senses in the future” (Franklin and Graesser, 1997).

A central feature of LIDA is a “cognitive cycle” wherein learning, perception, and action occur. The core part of the cognitive cycle is a global broadcast of information across the mind. The first step in the cycle is input, either exogenous (e.g., sensory input) or endogenous (e.g., memory), that results in perception and/or understanding of the current situation. Second, in the attention phase, information that has reached a certain level of salience is broadcast. Third, this broadcasted information results in learning and/or action.

Cognitive cycles can overlap and it is possible for actions to occur both asynchronously and without rising to the level of consciousness (i.e., without being globally broadcast). While the majority of LIDA research thus far has focused on fleshing out the cognitive cycle, recent developments have included cases that require multiple cognitive cycles, including distal intentions and narratives (Kronsted et al., 2021), the body schema (Neemeh et al., 2021), and smooth coping (Kronsted et al., 2022). Modeling musical improvisation will likewise require multiple cognitive cycles.

**FIGURE 2:**
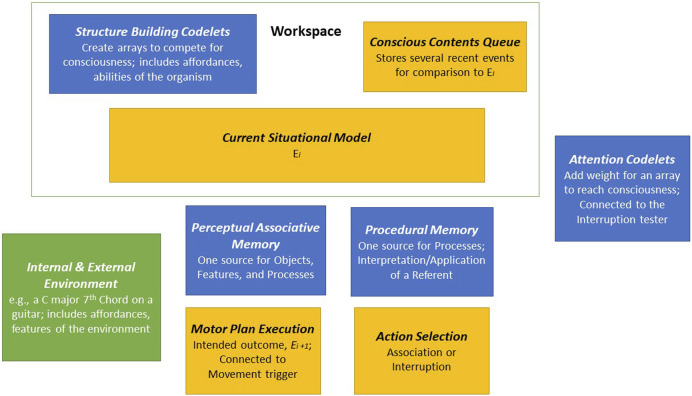
Musical Improvisation in LIDA–A Partial Conceptual Implementation.

Fully implementing Pressing’s model in LIDA is outside the scope of this current work, but three points can be said about a partial implementation. First, modeling expertise in musical improvisation will require further development of codelets. Codelets, especially for attention and structure-building, are important parts of LIDA. They fulfill tasks such as bringing together different types of information or raising relevant information to the level of consciousness. In addition, work on LIDA has considered a specific role for some structure-building codelets in creating affordance links (Franklin et al., 2016; Section 5.3.2). Second, further developing the Perceptual Associative Memory and Procedural Memory modules will be important for modeling skills in musical improvisation, especially involving a referent. Third, the Conscious Contents Queue, Action Selection and Motor Plan Execution modules will be essential parts of grounding any model of improvisation. Part of developing these modules will connect to how improvisation occurs with and without consciousness. Figure 2.

One may wonder why I appeal to LIDA, rather than another Artificial General Intelligence (AGI) model. On one hand, it is useful to develop as many cognitive architectures as possible. On the other, LIDA offers several unique aspects that have been underexplored in AGI. For example, LIDA’s focus on AAs means that it does not reduce improvisation to a problem-solving task, since the explorations, sensations, and alterations that are part of being an AA covers a wide swath of why we improvise. The central focus on affect, emotion, and consciousness also makes LIDA an important architecture to use and further develop; indeed, while affect and emotion are an essential part of all human cognition, they play an extremely strong role in music (Schiavio et al., 2017; Van der Schyff and Schiavio, 2017; Novelli and Proksch, 2022).

## 4 An ecological description and musical affordances in improvisation

Love (2017) provides an ecological description of jazz improvisation. His paper begins with a concern of oversimplification for computational models. Specifically, while simplification is needed for computational purposes, it does not match actual human improvising (Love, 2017, 32). He raises a connected worry about any neat distinctions between input, processing, and output, since cognition in the world is neither that simple nor clean. In turn, Love develops his description by reformulating ideas from Pressing’s model, including the referent, the role of memory and learning, and the temporal coordination among soloists and ensembles. I shall focus on the referent and memory/learning in what follows.

First, according to Pressing, a benefit of the referent is to simplify improvisation. Following chord changes allows a soloist to focus on other aspects of creativity, for instance. Love grants this simplification as important. However, he notes that a referent may also make improvising more difficult “by introducing the possibility of failure, or, if we prefer, shrinking the set of actions that count as ‘success’” (Love, 2017, 34). This difficulty is connected to the idea that improvisation is a form of navigation across a terrain, rather than abstract problem-solving. Any solo navigational difficulty is moreover amplified within collective forms of musical improvisation, where ensembles support or challenge the soloist navigating their environment (Linson and Clarke, 2017).

Second, instead of long-term memory, Love’s ecological description focuses on perceptual process and affordances. An affordance is a relational property between the abilities of an organism and features of the environment (Chemero, 2009). For instance, the affordance “climbable” is present if an organism is able to scale vertical surfaces in the environment; a specific “climbable” surface for a human may not exist for a dog, just as a professional climber may find surfaces “climbable” that are impossible for a novice. There is also an important role for affordances in the design of everyday objects, as emphasized by engineer and design expert Norman (2013).

Following philosophers Rietveld and Kiverstein. (2014), I suggest that affordances are found in a landscape for a form of life. Any situation will include a salient field of affordances within the overall landscape (Einarsson and Ziemke, 2017). The form of life is the focal point since it helps make sense for how affordances come into existence and how they persist over time.

Finally, Love cites several additional pieces of evidence about memory, including worries about the overemphasis of memory across perception and the fact that much of what an improvisor does is connected to how they perceive and navigate an improvisation, instead of using an abstract store of representations separate from perception itself (Love, 2017, 38–40).

I have been calling Love’s work a description, rather than a theory or model. The reason why, as Love himself notes, is his account lacks core aspects of a theory, especially falsifiability (Love, 2017, 31). By connecting Pressing and Love directly to LIDA, I suggest that we will at least take steps in the direction of a developed theory, even though I do not provide that fully developed theory here. Furthermore, LIDA is integrally connected to several additional theoretical and empirical commitments, most notably Bernard Baars’ Global Workspace Theory (GWT) (Franklin et al., 2016; Section 4.4). These connections help support the idea that my proposed account will be part of a general theory, not merely a description.

## 5 Discussion

There are two main points I will consider in this discussion: First, does my model call for a major reformulation or minor refocus in Pressing’s account? Second, is LIDA able to fully capture the affordances that are essential to an ecological account of improvisation?

For the first point, I suggest that the answer will partially rest on the extent to which Pressing’s model contains the ecological aspects highlighted by Love. At first glance, Pressing’s model already includes an explicit role for performer-environmental interaction. He likewise emphasizes a refinement of perception and action in musical performance, in part, by learning to discern invariant structures of the environment. Pressing introduces invariants in ways that are similar to affordances. When discussing “arrays” of objects, features, and processes, for instance, he notes that “the answer given here is based on an ecological perspective, which considers that the capacity to extract or create such arrays is neurologically innate, but that they are only brought into being by interaction with the environment” (Pressing, 1988, 161). We find a similar situation with other parts of the knowledge base, notably “perceptual strategies” (1998, 53), which implies that perception does not collapse into another form of memory.

The two possible results to the first point are either (1) Love is offering a minor shift in emphasis rather than a major reformulation or (2) Love’s reformulation comes mainly from worries that Pressing’s account fails to capture other essential aspects of affordances and/or the environment beyond invariance. While I am not committed to either of these interpretations, the best likelihood for (2) would come from the subjective component of affordances and their “ambivalent relationship to rules” (Love, 2017, 34). (2) may also be supported by Pressing’s explicit rejection of “the organizational invariant approach” as a satisfactory theory for modeling action and improvisation (1988, 133–4).

This rejection of an organizational invariant approach raises a concern that Pressing’s account is not about the perception of affordances because it requires a comparison of sensation to pre-existing knowledge, instead of the agent adapting to affordances in the environment. One reply to this concern is to grant that Pressing’s approach and affordances are more opposed than I heretofore implied, with substantial work required to reconcile them, if doing so is even possible. A second reply is to consider recent work on neural resonance as a way to account for organism abilities and the knowledge base in an ecological manner (Fuchs, 2018; Raja, 2018; Ryan and Gallagher, 2020; Shepard, 1984; for neural resonance and neurodynamics in musical perception, see Large, 2010). This second option, if correct, still requires more work, but it would be closer to a slight refocus over radical reformulation.

For the second main point, one may be concerned with the vast assortment of memory modules in LIDA with limited cases of distinctly perceptual processes. Another issue may be that the affordances introduced so far in LIDA are not adequately relational. To address these worries, I will say a few words on the connection between LIDA and embodied cognition, along with the importance of looking at the implementation of the LIDA model in LIDA agents as part of the modeling process.

Franklin et al. (2016) argue that LIDA is “resonant with the core ideas of the embodied, situated, and enactive views” (2016, Section 4.2), with situated cognition being close to the core commitments of ecological psychology introduced so far (i.e., the mutual influence and interaction of environment and organism in cognition). Three points of connection they discuss in detail are asynchrony, nonlinear dynamics, and Theta Gamma Coupling. Of primary note here is asynchrony. While much of the description of LIDA introduces the model as if it works in a serial fashion, that is only for ease of explanation. In practice, almost all of the processing in the LIDA architecture may occur in asynchronous fashion, which entails that the “LIDA model accommodates the possibility of algorithmic behavior more complex than that of a data pipeline in the information processing paradigm.” (Franklin et al., 2016 Section 4.9). Breaking down this pipeline is akin to what Love calls for when he questions the neat distinction of input-process-output.

Additionally, an implementation of LIDA in a LIDA agent is essential when considering the nature of affordances themselves. An affordance is a relational property between an organism and the environment. While certain abstract claims can be made about them, an affordance must be grounded in specific organisms acting in specific environments. Similarly, affordances in LIDA are partially constituted by implementation in an agent, rather than being fully, and solely, developed in the conceptual model. It may even be the case that using the model alone to tell us everything about affordances could be a category mistake.

## 6 Conclusion

I have proposed that a fruitful avenue for understanding musical improvisation will come from a combination of Pressing’s model, taken under Love’s ecological description, and the Learning Intelligent Decision Agent (LIDA) cognitive architecture. My theory includes at least the following three points, which are open to further exploration, refinement, or change as additional research is conducted.

• Insights from a traditional cognitive model and an ecological description of that model can be fruitfully combined within an AGI architecture;
•While this combination draws on existent theoretical components, it also opens up important theoretical and empirical questions for the future (e.g., how can we build an improvising LIDA robot? In what ways will it be unique and similar to existent improvising AI machines? How can we use the LIDA cognitive model and musical improvisation to test open questions about Global Workspace Theory and other accounts of consciousness? What aspects of improvisation occur within and outside of consciousness?);
• An account of musical improvisation is integrally connected to other forms of improvisation, and we cannot fully understand any of those activities in complete isolation.

Moving forward, it will likely be important to further nest the aforementioned approaches within even broader theoretical frameworks, such as the Skilled Intentionality Framework (Rietveld, Denys and Van Westen, 2018) or the Thinking Through Other Minds and Cultural Affordances Frameworks (Ramstead, Veissière and Kirmayer, 2016; Veissière et al., 2020). Integrating more interdisciplinary sources and artistic research will be essential as well, including work by Derek Bailey, Gary Peters, Marcel Cobussen, and Harald Stenström (e.g., Bailey, 1992; Stenström, 2009; Cobussen, 2017; Peters, 2017). Such additions will further develop this model of musical improvisation and its related upshots.

## Data Availability

The original contributions presented in the study are included in the article/supplementary material, further inquiries can be directed to the corresponding author.

## Footnotes

1 It is important to note that Pressing’s model is best fit for “standard” jazz improvisation, especially in the context of either solo performance and/or group improvisations with a clear referent. While it is not limited to these contexts, we may need to consider different models (e.g., MacDonald and Wilson, 2020, esp. Chap. 4; Canonne and Garnier, 2011) for capturing “free” jazz performance or improvisation in other genres of music.
